# Potential Long-Term Complications of Endovascular Stent Grafting for Blunt Thoracic Aortic Injury

**DOI:** 10.1100/2012/897489

**Published:** 2012-04-01

**Authors:** Larry E. Miller

**Affiliations:** Miller Scientific Consulting, Inc., 26 Portobello Road, Arden, NC 28704, USA

## Abstract

Blunt thoracic aortic injury (BTAI) is a rare, but lethal, consequence of rapid deceleration events. Most victims of BTAI die at the scene of the accident. Of those who arrive to the hospital alive, expedient aortic intervention significantly improves survival. Thoracic endovascular aortic repair (TEVAR) has been accepted as the standard of care for BTAI at many centers, primarily due to the convincing evidence of lower mortality and morbidity in comparison to open surgery. However, less attention has been given to potential long-term complications of TEVAR for BTAI. This paper focuses on these complications, which include progressive aortic expansion with aging, inadequate stent graft characteristics, device durability concerns, long-term radiation exposure concerns from follow-up computed tomography scans, and the potential for (Victims of Modern Imaging Technology) VOMIT.

## 1. Background

Blunt thoracic aortic injury (BTAI) is a leading cause of mortality in trauma patients, accounting for 8,000 deaths per year in North America [1]. Automobile accidents are responsible for most of these injuries [1, 2] although BTAI from autopedestrian collisions [3], aircraft accidents [4], falls [5], and crush injuries [6] has been reported. Rapid deceleration causes shearing forces at the aortic isthmus, acute diaphragmatic compression, aortic torsion, and aortic compression between the spine and sternum. Deceleration-induced BTAI typically results in a transverse aortic tear near the ligamentum arteriosum [2, 7] ranging in severity from a partial circumferential intimal tear that may spontaneously heal with no intervention to complete aortic transection resulting in rapid exsanguination and death. Approximately, 80–90% of BTAI victims die before reaching the hospital [6, 8]. Of the patients who arrive at the hospital alive, mortality is 65% unless aortic repair with open surgery or thoracic endovascular aortic repair (TEVAR) is performed [9]. 

Until recently, surgical repair of the aorta was the standard of care for BTAI management. Most cases involve surgical interposition of a synthetic graft with active bypass to achieve distal aortic perfusion although simple repair can be undertaken for minimal aortic injuries. However, surgical aortic repair is associated with significant mortality and morbidity. Since the first report of TEVAR for treatment of BTAI in 1997 [10], this procedure has become standard of care at many sites due to the avoidance of left thoracotomy, aortic cross-clamping, single-lung ventilation, and systemic anticoagulation and because of the superior short-term outcomes relative to open surgery. Currently, no endograft has been approved for the treatment of BTAI, and, therefore, experience with these devices is limited to clinical trials at centers of excellence or to off-label use.

## 2. Treatments for Blunt Thoracic Aortic Injury

The clinical benefit of aortic repair following BTAI, regardless of the method used, is obvious. With nonsurgical management of BTAI, mortality is approximately 1% per hour over the first 48 hours [6]. Aortic intervention significantly alters the natural history of BTAI with TEVAR offering short-term benefits over surgical repair. Hoffer et al. [11] reviewed 19 studies that compared TEVAR to open surgery for treatment of BTAI. TEVAR was associated with lower risk of mortality (OR = 0.43) and paraplegia (OR = 0.30). A meta-analysis by Xenos and colleagues [12] reported similar outcomes favoring TEVAR with an odds ratio of 0.44 and 0.32 for mortality and paraplegia, respectively. The following year, this same group updated these findings with similar results [13]. Tang et al. reported that TEVAR reduced mortality by 50% versus open surgery and resulted in no paraplegia (versus 5.6% for surgery) [14]. Finally, in a prospective nonrandomized multicenter trial, TEVAR resulted in superior results versus open surgery in mortality (7.2% versus 23.5%) but no statistical difference in paraplegia (0.8% versus 2.9%) [15]. On balance, in unselected populations, TEVAR offers no survival advantage or reduction in paraplegia risk [16]. This observation may be attributable to the higher rates of complications with TEVAR for BTAI at less-experienced centers [15]. 

The primary goal in the treatment of a BTAI patient is survival, and, therefore, favorable short-term outcomes are imperative. However, comparatively less attention has been given to potential long-term outcomes of TEVAR for BTAI. To date, no trials of TEVAR for BTAI have reported long-term clinical or radiographic outcomes. This paper will focus on the potential long-term complications in patients treated with TEVAR for BTAI, including progressive aortic expansion with aging, inadequate stent graft characteristics, device durability concerns, long-term radiation exposure concerns from follow-up computed tomography scans, and the potential for VOMIT (Victims of Modern Imaging Technology).

## 3. Potential Complications of Endovascular Stent Grafting for Blunt Thoracic Aortic Injury

### 3.1. Aortic Expansion over the Lifespan

The healthy thoracic aorta increases in diameter by approximately 1.5 mm per decade and, therefore, could theoretically grow almost 10 mm from adolescence to old age [17]. Recommended sizing of stent grafts for BTAI ranges from 0 to 20% larger than the aortic diameter. Based on these guidelines, a 27 mm diameter stent graft could safely accommodate an aorta of 22.5 to 27 mm in diameter. However, if a patient with a 24 mm diameter aorta is treated with a 27 mm device, the aorta may expand beyond the diameter of the stent graft in approximately 20 years, assuming the stent graft did not influence aortic growth. This degree of expansion would greatly increase the risk for device collapse, migration, and/or endoleak and, in the absence of definitive surgical repair, may require multiple TEVAR reinterventions over a lifetime to account for this progressive growth. Forbes and coworkers reviewed computed tomography scans from 21 patients who underwent TEVAR for BTAI and had at least 1-year follow-up imaging. Of concern, the aortic diameter immediately distal to the left subclavian artery increased at a rate of 0.8 mm per year over a mean 2.6 year follow-up period [18]—a rate of aortic expansion 5-fold faster than in the healthy aorta. The behavior of the aorta beyond this period is unknown, but these data further highlight the need for regular surveillance in these patients. Concerns around aortic growth may be somewhat tempered by the fact that placement of endografts results in fibrous reactions around the graft, which promotes aorta-to-graft adherence [17, 19]. 

### 3.2. Inadequate Stent Graft Characteristics

Advancements in stent graft technology have severely lagged behind the widespread off-label adoption of TEVAR for treatment of various aortic pathologies. Of the three thoracic stent grafts currently approved for use in the United States (Medtronic Talent, Cook TX2, and Gore TAG), none is indicated for use in the trauma patient. Furthermore, since these devices were approved for use in patients with degenerative aortic disease, the characteristics of current devices are less than ideal for BTAI patients. The ideal trauma-specific device would accommodate delivery through small access vessels and deployment in a small-diameter aorta with tight curvature of radius in the aortic arch and relatively higher pulsatile compliance and flow velocity.

The mean aortic diameter adjacent to a BTAI is 19 mm with the smallest reported to be 14 mm [2]. However, available stent grafts cannot accommodate the entire range of aortic diameters in the typical BTAI patient. Consequently, many BTAI patients are either denied TEVAR due to small aortic diameter or are treated with excessively oversized, off-label devices. The tight angulation of the aortic arch in the young trauma patient poses additional challenges to correct endograft placement. Since most BTAIs occur near the ligamentum arteriosum with a mean 5.8 mm length between the injury and the left subclavian artery ostium when measured along the lesser curve [2], the proximal landing zone of the endograft is generally just distal to the left subclavian artery ostium or, in some cases, distal to the left common carotid artery necessitating coverage of the left subclavian artery. The combination of a landing zone in a tight aortic arch with a relatively inflexible stent graft can result in bird beaking, a phenomenon characterized by malapposition of the stent graft with the aortic wall, which is associated with a 64% endoleak rate and a known risk factor for subsequent device collapse [20].

Device collapse is a phenomenon observed in 1% to 19% of TEVAR repairs for BTAI and is primarily attributable to excessive device oversizing combined with a tight aortic curvature radius [21]. In fact, the typical collapsed endograft is oversized by 27 ± 12% [22]. While typically noted within 30 days of treatment, device collapses have been reported after 3 years after TEVAR [21]. Device collapses have been most commonly reported with the Gore TAG device with 169 reported collapses, of which 72% required reintervention and 8% resulted in death, according to the manufacturer's 2011 Annual Clinical Update [23]. Further, it has been suggested that the radial strength of the Gore TAG device is insufficient to prevent device infolding when the inferior edge of the device protrudes into the aortic lumen, particularly with an oversized device in a small aorta [24]. In contrast, the Talent and TX2 devices are purported to have relatively superior radial strength with extremely rare device collapses reported [21, 24, 25]. These data suggest that endograft selection may influence treatment outcomes of BTAI. Clinical trials are underway with trauma-specific devices that have more sizing options, better radial strength, and better conformability to the aortic arch, thereby potentially minimizing the bird beaking effect.

### 3.3. Device Durability Concerns

Clinical trials of endovascular stent grafts regulated by the Food and Drug Administration typically mandate patient followup for 5 years after treatment. Pulsatile fatigue testing of thoracic endografts is generally simulated over 400 million cycles, equivalent to 10 years in vivo. These durations are adequate for the study of thoracic aortic aneurysm since the typical patient lifespan after TEVAR is 7 years [26]. However, for the BTAI patient treated with TEVAR with an expected lifespan of several decades, long-term device durability becomes a major concern and current stent graft evaluation methods are likely inadequate. To date, the longest reported mean follow-up duration following TEVAR for BTAI is 8.7 years [27] with no significant survival advantage reported over open surgery through 4 years (11% versus 16%, *P* = 0.7) [28]. Beyond this period, clinical outcomes are unknown. Stent graft manufacturers should consider extended durability testing for trauma-specific stent grafts commensurate with extended posttreatment patient life spans. Furthermore, regulators should consider mandating longer follow-up periods for clinical trials of TEVAR for BTAI in order to collect, at a minimum, mortality and device-specific data. 

### 3.4. Excessive Cumulative Radiation Exposure

Helical CT scans of the chest have become the diagnostic tool of choice for patients with suspected deceleration injury. A single chest CT exposes a patient to approximately 7 mSv of ionizing radiation. For reference, a single exposure to 10 mSv of ionizing radiation will ultimately cause cancer in 1 out of 1,000 people [29]. Assuming a 40-year-old trauma patient with a life expectancy of 30 additional years and annual follow-up examinations with contrast-enhanced chest CT, the cumulative CT effective dose would be 210 mSv, significantly increasing malignancy risk. Furthermore, the risk for radiation-induced cancer is greater in a younger patient (e.g., typical trauma victim) versus an older patient [29]. Overall, the benefit of lifelong annual CT scans for assessing endograft status may be, to a degree, offset by the elevated risk of cancer due to high lifelong exposure to ionizing radiation.

### 3.5. Potential for VOMIT

Concomitant with the advent of high-resolution helical CT scanning for the diagnosis of suspected BTAI, identification of minimal aortic lesions has become increasingly prevalent. Approximately 10% of patients with BTAI experience minimal aortic injuries that result in focal intimal tears with no or little involvement of the media [30]. However, 21% of BTAI patients with minimal aortic injuries undergo TEVAR [15] despite the clinical practice guidelines of the Society of Vascular Surgery to the contrary [31]. In vitro and in vivo animal studies have demonstrated that arterial injuries limited to the intima and inner media heal completely with no intervention [32–35]. Malhotra and colleagues reported no aortic ruptures in 9 patients with minimal traumatic aortic injuries who underwent nonoperative management [30]. On balance, the definition of minimal is ambiguous and the natural history of these injuries may include pseudoaneurysm development in up to 50% of cases [30]. Nonetheless, there is growing concern that minimal aortic injury identified on CT combined with the widespread enthusiasm for stent grafting may result in unnecessary TEVAR treatment. The subset of patients who are unnecessarily subjected to TEVAR in the presence of a minimal BTAI, otherwise referred to as VOMIT (Victims of Modern Imaging Technology) [36], are subjected to unnecessary procedural- and device-related risks given the overall favorable prognosis of untreated minimal aortic injury. While not a long-term complication *per se*, these patients are prone to all long-term device risks previously mentioned (including a 20% risk of device-related complication in the postoperative period [15]) when, in fact, they arguably could have been successfully treated with nonoperative measures.

## 4. Summary

Evaluation of long-term complications following TEVAR for BTAI is best managed by regular clinical and radiographical patient followup. However, the typical BTAI patient is notoriously unreliable in attending scheduled follow-up visits [37–39]. In fact, one study of TEVAR for BTAI reported that 70% of patients did not return for follow-up imaging after hospital discharge despite patient education before discharge and aggressive attempts at patient contact after discharge [40]. Therefore, the potential for long-term complications of TEVAR for treatment of BTAI may be further exaggerated by the lack of ongoing medical surveillance. Even as stent graft manufacturers continue efforts to develop trauma-specific endografts, the issues of continued aortic expansion, unknown device durability, excessive radiation exposure, and the potential for VOMIT remain. In conclusion, despite the unmistakable short-term benefit of TEVAR in the BTAI patient, there is a largely unrecognized potential for serious long-term complications with this procedure.
